# Protein Biomarker Identification for the Discrimination of *Brucella melitensis* Field Isolates From the *Brucella melitensis* Rev.1 Vaccine Strain by MALDI-TOF MS

**DOI:** 10.3389/fmicb.2021.712601

**Published:** 2021-10-22

**Authors:** David Kornspan, Holger Brendebach, Dirk Hofreuter, Shubham Mathur, Shlomo Eduardo Blum, Marcelo Fleker, Svetlana Bardenstein, Sascha Al Dahouk

**Affiliations:** ^1^Department of Bacteriology, Kimron Veterinary Institute (KVI), Bet Dagan, Israel; ^2^Department of Biological Safety, German Federal Institute for Risk Assessment (BfR), Berlin, Germany; ^3^Department of Serology, Kimron Veterinary Institute (KVI), Bet Dagan, Israel

**Keywords:** *Brucella melitensis*, MALDI-TOF MS, diagnostic, biomarker, vaccine, *in silico* proteomics

## Abstract

*Brucella melitensis* Rev.1 is a live attenuated vaccine strain that is widely used to control brucellosis in small ruminants. For successful surveillance and control programs, rapid identification and characterization of *Brucella* isolates and reliable differentiation of vaccinated and naturally infected animals are essential prerequisites. Although MALDI-TOF MS is increasingly applied in clinical microbiology laboratories for the diagnosis of brucellosis, species or even strain differentiation by this method remains a challenge. To detect biomarkers, which enable to distinguish the *B. melitensis* Rev.1 vaccine strain from *B. melitensis* field isolates, we initially searched for unique marker proteins by *in silico* comparison of the *B. melitensis* Rev.1 and 16M proteomes. We found 113 protein sequences of *B. melitensis* 16M that revealed a homologous sequence in the *B. melitensis* Rev.1 annotation and 17 of these sequences yielded potential biomarker pairs. MALDI-TOF MS spectra of 18 *B. melitensis* Rev.1 vaccine and 183 Israeli *B. melitensis* field isolates were subsequently analyzed to validate the identified marker candidates. This approach detected two genus-wide unique biomarkers with properties most similar to the ribosomal proteins L24 and S12. These two proteins clearly discriminated *B. melitensis* Rev.1 from the closely related *B. melitensis* 16M and the Israeli *B. melitensis* field isolates. In addition, we verified their discriminatory power using a set of *B. melitensis* strains from various origins and of different MLVA types. Based on our results, we propose MALDI-TOF MS profiling as a rapid, cost-effective alternative to the traditional, time-consuming approach to differentiate certain *B. melitensis* isolates on strain level.

## Introduction

Brucellosis is a global zoonotic disease affecting domestic and wild animals as well as humans (Pappas et al., 2006). Three out of twelve currently known *Brucella* species are responsible for most of the reported human brucellosis cases, namely *B. melitensis*, primarily transmitted from sheep and goats, *B. abortus* from cattle, and *B. suis* from swine (Hull and Schumaker, 2018). Ovine and caprine brucellosis are endemic throughout the Middle East as well as in many countries of Africa, Asia, and Latin America (Rossetti et al., 2017). In these regions, efforts are undertaken to control brucellosis by the vaccination of sheep and goats.

The most common vaccination policy for small ruminant livestock against *B. melitensis* infections is the application of the live attenuated *B. melitensis* Rev.1 strain to female animals aged between 2 and 6 months (Banai, 2002). This procedure has proven to be protective and to reduce abortions in treated animals, but may be contraindicated in females that are vaccinated during their last trimester of pregnancy (Banai, 2002). Although attenuated, the *B. melitensis* Rev.1 strain is still capable to infect humans and cause disease, either through the consumption of contaminated milk from vaccinated animals or by accidental exposure during the vaccination procedures (Arapovic et al., 2020). Hence, laboratory methods that can easily distinguish *B. melitensis* field strains from the vaccine strain are relevant for (i) effective brucellosis control programs, (ii) for epidemiological surveillance, and (iii) for outbreak clarification.

*Brucella melitensis* Rev.1 possesses several characteristics, including streptomycin resistance and a distinct dye sensitivity pattern, which enables its discrimination from field strains using bacteriological tests (Elberg and Meyer, 1958). However, atypical *B. melitensis* biovar 1 field and Rev.1 vaccine isolates have been described, which may lead to misinterpretations (Banai et al., 1990; Banai, 2002; Lucero et al., 2006). Moreover, *B. melitensis* Rev.1 induces like other *B. melitensis* strains the production of antibodies directed against its smooth lipopolysaccharide (LPS). This property interferes with serological testing for brucellosis due to cross-reactivity between the smooth LPS of the vaccine strain and other smooth *Brucella* species or the LPS of widespread Gram-negative pathogens such as *Yersinia enterocolitica* and *Salmonella* spp. (Corbell, 1975). Hence, more robust molecular markers are needed, and a molecular screening method has been developed based on the *Pst*I site polymorphism in the *Brucella omp*2 gene of the *B. melitensis* Rev.1 vaccine strain, which allows its differentiation from *B. melitensis* field isolates (Bardenstein et al., 2002). In addition, *rpsL*-directed PCR-RFLP and multiplex PCR assays have been established to discriminate *B. melitensis* biovar 1 wild-type strains from *B. melitensis* Rev.1 (Cloeckaert et al., 2002; Garcia-Yoldi et al., 2006).

Similar to PCR-based microbial diagnostics, matrix-assisted laser desorption/ionization–time of flight mass spectrometry (MALDI-TOF MS) profiling has emerged as a rapid and cost-effective laboratory method to identify bacteria in recent years (Croxatto et al., 2012). Rigorous preprocessing and generous peak binning during spectra creation for MALDI-TOF MS platforms, like Bruker Biotyper, Vitek MS or Andromas lead to robust identification performances for many bacteria at genus and even at species level (Clark et al., 2013). However, strain identification relies on discriminating strain-specific differences in the proteome under the constraints of MALDI-TOF MS. These subtle differences are caused by genomic alterations, i.e., indels, frameshifts, non-synonymous single nucleotide polymorphisms or pseudogenization that manifest as a change in abundance or result in an altered amino acid sequence and post-translational modifications of a protein. While pseudogenization or changes in steady-state levels of proteins may be detected by modified peak intensities, changes in the molecular composition of a protein may lead to a unique mass shift of its corresponding peak, oftentimes referred to as a biomarker. Species- and even strain-specific biomarkers have been reported for the MALDI-TOF MS-based identification and differentiation of various bacterial pathogens, like *Haemophilus* spp., *Helicobacter pylori*, *Campylobacter* spp., *Yersinia enterocolitica* or methicillin-resistant *Staphylococcus aureus* (Sandrin et al., 2013). MALDI-TOF MS has also been applied for the identification of *Brucella*. However, classical *Brucella* species are highly homologous (Hoyer and Mccullough, 1968), which is why commercially available reference libraries have shown shortcomings in the reliable classification of *Brucella* beyond genus level (Ferreira et al., 2010; Cunningham and Patel, 2013; Tracz et al., 2016) or the differentiation of closely related *Ochrobactrum* species (Poonawala et al., 2018), recently reclassified as *Brucella* spp., for example *B. anthropi* and *B. intermedium* (Hordt et al., 2020). However, the generation of in-house reference libraries of MALDI-TOF MS spectra may allow for correct identification of *Brucella* species and some of their respective biovars (Lista et al., 2011; Karger et al., 2013; Mesureur et al., 2018; Da Silva et al., 2020).

Our study aimed to establish a novel MALDI-TOF MS-based diagnostic approach that facilitates the rapid differentiation of the *B. melitensis* Rev.1 vaccine strain from *B. melitensis* field isolates. We took advantage of a comparative *in silico* proteomics analysis and a comprehensive in-house library of MALDI-TOF MS spectra to identify specific protein biomarkers for the resolution of *B. melitensis* isolates on sub-species level.

## Materials and Methods

### *In silico* Proteome Comparison

Complete genomes of *B. melitensis* biovar 1 strain 16M, GCF_000007125.1_ASM712v1 (Delvecchio et al., 2002) and GCF_000740415.1_ASM74041v1 (Minogue et al., 2014), as well as *B. melitensis* biovar 1 strain Rev.1, GCF_002953595.1_ASM295359v1 (Salmon-Divon et al., 2018), were retrieved from the National Center for Biotechnology Information (NCBI^1^). The genome sequences were submitted to the Pathosystems Resource Integration Center (PATRIC^2^) for RASTtk annotation to augment the protein features with genus-specific “local protein family properties” (called PLfam) (Brettin et al., 2015).

The newly annotated protein coding sequences (CDS) were treated as strings and the subset


$$\left(GCF_{000007125.1}\cup GCF_{000740415.1}\cup GCF_{002953595.1}\right)\backslash \left(G\,C\,F_{000007125.1}\cap G\,C\,F_{000740415.1}\cap G\,C\,F_{002953595.1}\right)$$

was filtered for the single presence of PLfam identifiers in all three PATRIC annotation feature tables (Supplementary Table 1). In a second filter step, only PLfam IDs were kept that shared the same sequence in the *B. melitensis* 16M genomes but had a divergent one in *B. melitensis* Rev.1 due to amino acid substitutions with a small mass shift (Δmass ± 130 Da) (Supplementary Table 2). Proteins in the target range of MALDI-TOF MS, i.e., with exact masses between 2,000 and 20,000 Da, were short-listed in Table 1 and annotated with UniProt identifiers of the *B. melitensis* strain 16M proteome (ID: BRUME). UniProt entries were screened for potential post-translational modifications (PTM), and where applicable, included into the calculation of the exact mass with the R package SeqinR v3.6.1 (Charif and Lobry, 2007). The frequent event of protein N-terminal methionine excision (NME, Δmass = −131.2 Da) was always considered, as well as beta-methylthiolation (βMeS, Δmass = +46.1 Da) for ribosomal protein S12.

**TABLE 1:** Short-listed proteins with discriminatory mass differences in the working range of MALDI-TOF MS.

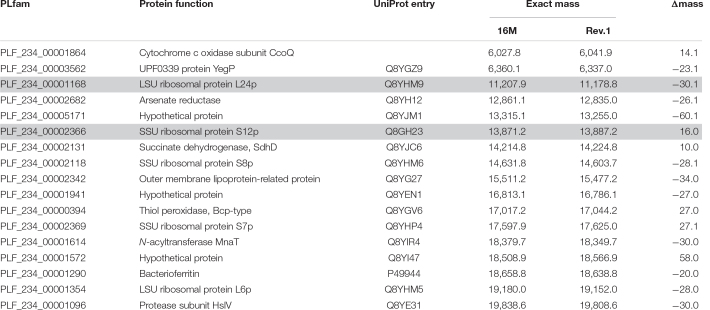

*PLfam, PATRIC genus-specific families identifier; masses in Dalton.*

### Bacterial Strains and Culture Conditions

Bacteria analyzed in the present study were 18 *B. melitensis* Rev.1 vaccine isolates (including the original Elberg Rev.1 vaccine strain, passage no. 101) and 183 *B. melitensis* field isolates from human, cattle, sheep and goats in Israel (Supplementary Table 3). All *Brucella* strains were obtained from the collection of the Kimron Veterinary Institute (KVI) in Bet Dagan, Israel, and were cultured for 48–72 h on tryptic soy agar (TSA) plates at 37°C under 5% CO_2_. All bacteria were characterized using standard methods: growth on TSA plates with penicillin G and streptomycin, dye sensitivity (thionine, fuchsine), H_2_S production, urease activity, and agglutination with mono-specific anti-M and anti-A serum. *Brucella melitensis* Rev.1 isolates were verified by *omp*2 PCR and *Pst*I digestion of the amplicon (Bardenstein et al., 2002). We prepared biological triplicates of *B. melitensis* Rev.1 vaccine isolates but only produced single preparations of the Israeli *B. melitensis* field isolates. Bacteria were grown with shaking in 10 ml of a tryptic soy broth (TSB pH 7.3) at 37°C for 24 h (OD_600_∼0.3–0.4), centrifuged at 7,000 × *g*, washed with PBS and resuspended in 300 μl PBS and 900 μl 100% ethanol. The bacterial solutions were left at room temperature for 48 h before 100 μl were plated on TSA plates for sterility testing. The work on live agents was performed at the KVI biosafety level 3 facility. Inactivated *Brucella* samples were split into aliquots and stored at −20°C for on-site use or shipping on dry ice to the German Federal Institute for Risk Assessment (BfR) in Berlin, Germany.

### MALDI-TOF

#### Spectra Acquisition

At both institutes, samples were independently prepared for mass spectrometry by ethanol-formic acid extraction according to manufacturer’s instructions before being spotted on a 96-spot steel plate target and covered with alpha-cyano-4-hydroxy-cinnamic acid (HCCA) matrix solution (Bruker Daltonik GmbH, Bremen, Germany). Mass spectra were measured using a microflex LT MALDI-TOF MS system (Bruker Daltonik) operated by the Biotyper automation software flexControl (v3.4.135.0, Bruker Daltonik). To increase data robustness, twelve technical replicate spectra were acquired from four different target spots using the recommended instrument settings for bacterial identification (linear positive ion detection mode, 60 Hz laser frequency, 20 kV acceleration voltage, 18.1–18.2 kV IS2 voltage). Spectra were initially analyzed at BfR using the Bruker Biotyper software (v3.1) with MSP library version MBT_7311 (7311 entries), the Security-Relevant (SR) Database (104 entries) and a customized in-house database to confirm identification as *B. melitensis*.

#### Data Analysis

Raw spectra data from the Bruker microflex MS instruments were imported into the statistical computing environment “R” (v3.6.3) and analyzed with the R packages MALDIquantForeign (v0.12) and MALDIquant (v1.19.3) (Gibb and Strimmer, 2012). Raw spectra were preprocessed using default functions and parameters, i.e., square root transformation, smoothing, baseline removal and normalization. Spectra were combined by the function *averageMassSpectra*, first by averaging technical replicate spectra into a “sample spectrum” and second by averaging sample spectra of the same group into a “group spectrum.” The groups were defined by bacteriological classification of a sample as “*melitensis* Rev.1” or the field isolate outgroup “*melitensis*” as well as by the institute where protein extracts were subjected to MALDI-TOF MS, namely “BfR” or “KVI.” Two intermediary spectra alignment steps were applied in the preprocessing: technical replicate spectra were aligned against each other whereas sample spectra were aligned against 37 reference peaks derived from all available sample spectra (method = “strict,” minFrequency = 0.9, tolerance = 0.002) of this study (Supplementary Table 4). The reference peaks with a relative frequency of ≥ 90% were distributed in a mass interval between 3,100 and 11,500 Da. All graphical spectra representations (gel, spectra and peaks) were drawn with customized ggplot2 functions from the R package Tidyverse v1.3.0 (Wickham et al., 2019). A comparison of all sample spectra is shown in Supplementary Figure 1.

#### Biomarker Validation

Sample spectra from the groups “*melitensis* Rev.1” and “*melitensis*” were manually screened for peaks corresponding to exact mass value pairs of short-listed proteins (Table 1) from the *B. melitensis* Rev.1 and 16M proteomes, respectively. Potential mass shifts in the mass value pairs caused by protein-specific PTMs were also considered. Biomarker mass intervals and decision boundaries for Rev.1 classification were determined by visual inspection of the peak distribution in the vicinity of accurate masses.

### *In silico* Genotyping and Pan Proteome Sequence Analysis

The NCBI database was queried^3^ for all RefSeq annotated assemblies of the genus *Brucella* (accession date 2020-11-05). Genomic and protein FASTA files of 802 samples were downloaded. Remarkably, this dataset also contained samples from the former taxon *Ochrobactrum*, which were used for comparison. The collection of FAA files was loaded into R and a binary matrix with unique sequences, the genus pan-proteome, and their presence therein was built. We calculated Jaccard coefficients, performed hierarchical clustering by UPGMA and visualized the phylogram (Figure 6) with the R package ggtree v2.4.1 (Yu, 2020). Molecular *in silico* typing was performed with the software tools (i) ‘‘MLST’’^4^ written by Thorsten Seemann using a MLST scheme with nine loci (Whatmore et al., 2007) retrieved from PubMLST (Jolley et al., 2018) and (ii) “MLVA” written by David Christiany (Vergnaud et al., 2018) using the MLVA panel 1 with eight loci and typing groups according to MLVAbank^5^. Typing results were combined when either one method yielded a typing group or both were concordant (Supplementary Table 5). Each RefSeq assembly was screened for variants of the ribosomal protein L24 or S12 sequence of *B. melitensis* 16M with blastP (Supplementary Table 5). True hits were used for annotation of the phylogram (Figure 6).

**FIGURE 1:**
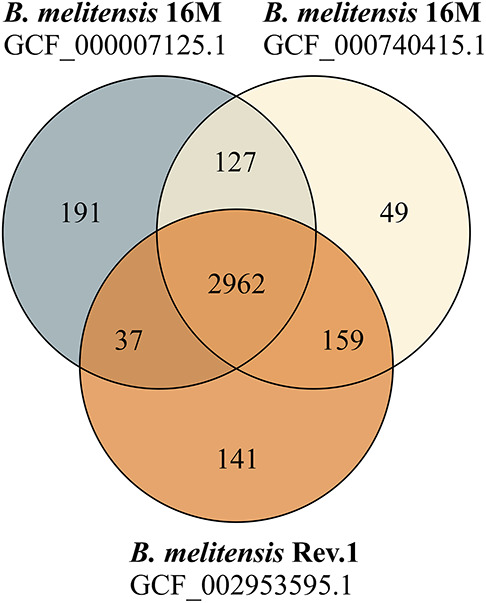
Venn diagram of shared and unique protein sequences found in RASTtk-annotated genomes. The protein-coding sequence sets of two *B. melitensis* 16M genomes (GCF_000007125.1: 3317 CDS; GCF_000740415.1: 3297 CDS) and one *B. melitensis* Rev.1 genome (GCF_002953595.1: 3299 CDS) were compared.

**FIGURE 2:**
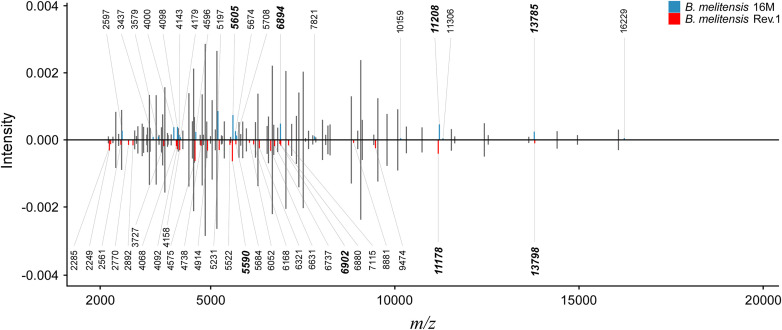
Peak comparison between *B. melitensis* 16M and *B. melitensis* Rev.1 strains. Shared peaks with the same *m/z* values are colored in gray without *m/z* label. Upper bar plot: *B. melitensis* 16M with unique peaks colored in blue. Lower bar plot: *B. melitensis* Rev.1 with unique peaks colored in red. For better comparison, *B. melitensis* Rev.1 intensity values were multiplied by –1. Bold *m/z* labels mark peak candidates that match mass difference and absolute mass of short-listed protein variants (Table 1). Peaks were averaged from sample spectra of three biological replicates with a peak frequency threshold of 50%.

**FIGURE 3:**
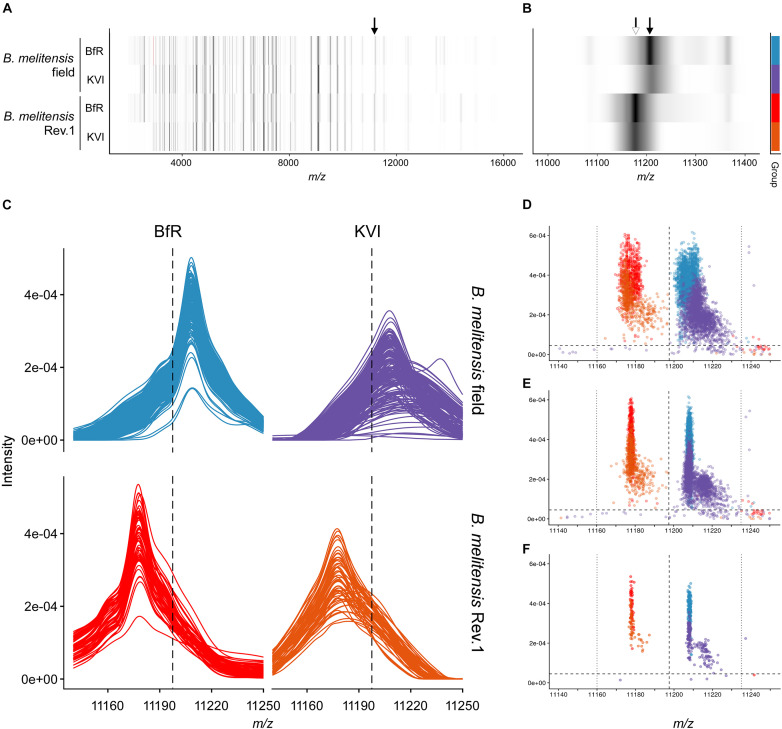
Group spectra and peak discrimination for the single charged ribosomal protein L24. **(A,B)** Gel view of group spectra derived from the *B. melitensis* field isolate group “*melitensis*” and the vaccine strain group “*melitensis* Rev.1” presented by institute “BfR” and “KVI,” in the full acquisition window **(A)** and magnified to the *m/z* range enclosing the exact masses of PLF_234_00001168, z = 1 **(B)**. Arrows point to accurate masses at 11,178 Da [white, (M_*Thr28*_+H)^+^ ion] and 11,208 Da [black; (M_Met28_+H)^+^ ion]. **(C)** Line graphs of aligned sample spectra shown by group and institute. **(D–F)** Scatterplot of peaks in the technical replicate spectra before **(D)** and after alignment **(E)** against the reference spectrum. (F) Peaks derived from aligned sample spectra. Intensity cut-off (horizontal dashed line) in panels **(C–F)**: 0.000045 AU; for mass cut-offs (decision window: vertical dotted lines, decision boundary: vertical dashed line) see Table 2.

**TABLE 2:** Statistics of strain discrimination by mass and intensity decision boundaries.

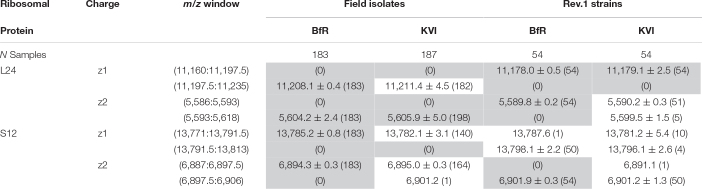

*Total number of peaks within boundaries (in parenthesis), their mean *m/z* and standard deviation are shown for field isolates and Rev.1 vaccine strains [format: 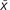 ± σ (*n*)]. Gray fields highlight biomarkers with 100% peak absence/presence and the discriminatory power of spectra in the respective institute. For some samples, we observed more than one peak in the *m/z* window. Ribosomal Protein L24: PLF_234_00001168; Ribosomal Protein S12: PLF_234_00002366. (PLfam, PATRIC genus-specific families; mass window unit: *m/z* in Dalton).*

**FIGURE 4:**
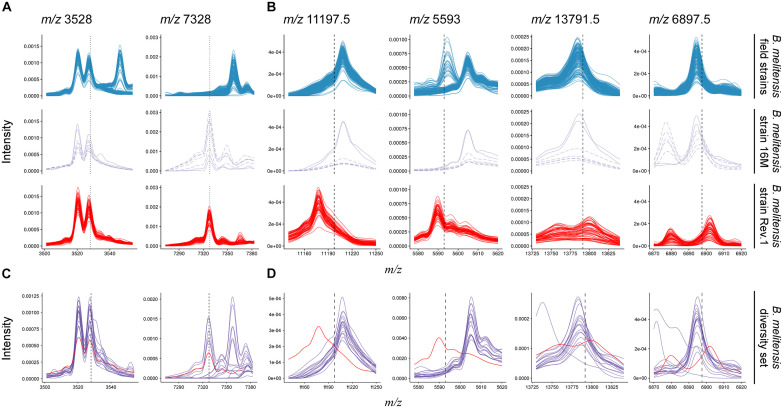
Comparison of *B. melitensis* field isolate (first row) and Rev.1 (third row) spectra against the type strain *B. melitensis* 16M (second row) and a diversity set of *B. melitensis* strains (fourth row) for selected masses. **(A)** Putative biomarkers (vertical dotted lines) described by Christoforidou et al. (2020). All spectra in this study displayed a peak at *m/z* 3,528. Most Rev.1 strains as well as the *B. melitensis* type strain 16M displayed a peak at *m/z* 7,328. **(B)** All biomarker decision boundaries (vertical dashed lines) in this study demarcate *B. melitensis* Rev.1 from *B. melitensis* 16M and field isolates. **(C,D)** Spectra from a diversity set of *B. melitensis* MVLA8 genotypes (purple) are plotted against a group spectrum of *B. melitensis* Rev.1 samples (red). For clarity, only spectra acquired at the BfR are shown.

**FIGURE 5:**
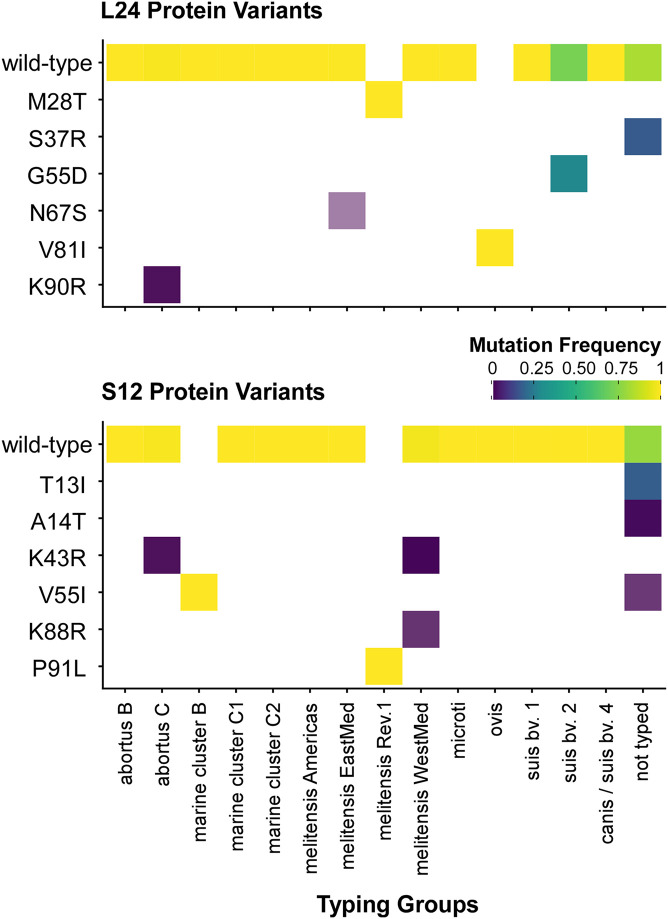
Distribution of ribosomal protein L24 and S12 variants in 802 *Brucella* genomes from the NCBI dataset. The heatmap coloring shows the frequency of each variant in the MLVA typing groups (white: no occurrence).

**FIGURE 6:**
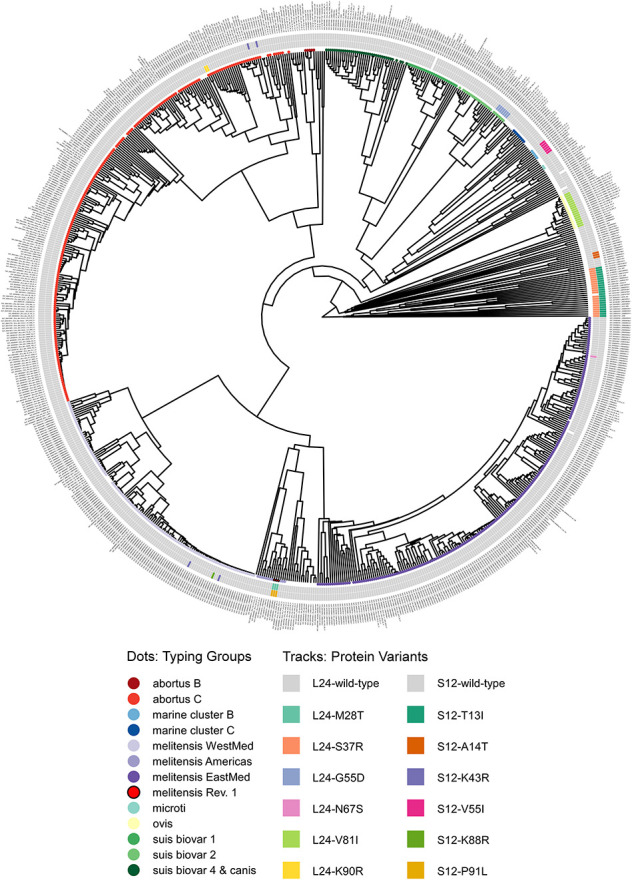
Phylogram of protein sequence presence in the NCBI pan proteome of the genus *Brucella*. Genomes were classified by *in silico* MLVA and MLST into typing groups (colored circles as leaves on the unrooted phylogram; white: no or non-congruent typing result). The tracks show ribosomal protein variants for L24 (inner) and S12 (outer) identified by blastP (white: no significant hit).

## Results

### *In silico* Identification of Biomarkers to Distinguish *Brucella melitensis* Rev.1 From the Reference Strain *Brucella melitensis* 16M

Our study aimed to identify MALDI-TOF MS biomarkers that allow for clearly distinguishing *B. melitensis* wild-type strains from the *B. melitensis* Rev.1 vaccine strain. Since MALDI-TOF MS for bacterial identification focusses on a narrow mass-over-charge (*m/z*) ratio ranging from 2,000 to 20,000, we first assessed a sequence-based *in silico* approach to find biomarkers. This method is feasible with respect to the limited number of expected divergent proteins useful for the discrimination of genetically closely related strains, such as *B. melitensis* Rev.1 and the reference strain *B. melitensis* 16M, both members of the same MLVA (Multiple-Locus Variable-number of tandem repeats Analysis) group “*melitensis* Americas” (Vergnaud et al., 2018). To this end, we compared the *in silico* translated open-reading frames (ORFs) of two different *B. melitensis* 16M complete genomes (Delvecchio et al., 2002; Minogue et al., 2014) against a complete *B. melitensis* Rev.1 genome (Salmon-Divon et al., 2018). To ease the matching of respective ORFs, all genomes were subjected to re-annotation by the RASTtk pipeline at PATRIC (Supplementary Table 1) and to a set analysis of their protein coding sequences. The resulting *in silico* core proteome shared by all three *B. melitensis* genome assemblies consisted of 2,962 translated coding sequences with identical amino acid sequences (Figure 1). Hence, about 90% of the protein sequences derived from the three *B. melitensis* genome sequences were not considered for our analysis. We further excluded 240 (191 + 49) proteins that differed between the two *B. melitensis* 16M annotations or were unique to either one and additional 196 (159 + 37) proteins identical between *B. melitensis* Rev.1 and only one of the two *B. melitensis* 16M annotations (Figure 1). Applying these filters, 113 out of 127 sequences identical in both *B. melitensis* 16M *in silico* proteomes matched with homologous sequences in the group of 141 annotated proteins specific for *B. melitensis* Rev.1. These 113 proteins harbor minor amino acid exchanges as a result of SNPs in their respective genes. Only few proteins matched the second filter criteria of bearing a mass within the working range of MALDI-TOF MS and a small but discernable mass difference.

The resulting short-list of 17 potential biomarker pairs (Table 1) is comprised of three hypothetical proteins, proteins with housekeeping enzymatic properties and five proteins (L24, L6, S12, S8, S7) of the 50S and 30S ribosomal subunits. Ribosomal proteins, highly abundant in bacterial cells, are well-known biomarkers for the identification and classification of bacteria by MALDI-TOF MS (Suarez et al., 2013).

### Ribosomal Proteins L24 and S12 Are Potential Discriminatory Marker Proteins to Identify the Vaccine Strain *Brucella melitensis* Rev.1

As a proof-of-concept for our bioinformatics approach, we screened an initial set of MALDI-TOF MS spectra from *B. melitensis* 16M and *B. melitensis* Rev.1 for the presence of the 17 group-specific *m/z* peak pairs and potentially post-translationally modified (PTM) variants thereof. For PTMs, we considered N-terminal methionine excision (NME, average Δmass = −131.2 Da), methylation (Δmass = 14.0 Da), acetylation (Δmass = 42.0 Da), and modifications noted in the UniProt database annotation of the respective proteins. Out of the 17 predicted biomarkers, a distinct peak pair in the sample spectra resembled the ribosomal protein L24 with averaged accurate masses at *m/z* 11,178 in the vaccine strain group “*melitensis* Rev.1” and *m/z* 11,208 in the reference strain group “*melitensis* 16M” (Figure 2). The exact mass difference of −30.1 Da between the ion [M_*Thr28*_+H]^+^*_*melitensis Rev.1*_* with *m/z* 11,178.8 and the ion [M_Met28_+H]^+^*_*melitensis 16M*_* with *m/z* 11,207.9 corresponded well to the observed peak shift. A further noticeable peak pair comprised the ribosomal protein S12 with averaged accurate masses at *m/z* 13,785 in the field strain group “*melitensis* 16M” and *m/z* 13,798 in the vaccine strain group “*melitensis* Rev.1.” The exact mass difference of +16.0 Da between the ion [M_Pro91_+H]^+^*_*melitensis 16M*_* with *m/z* 13,871.2 and the ion [M_Leu91_+H]^+^*_*melitensis Rev.1*_* with *m/z* 13,887.2 matched well to the observed peak shift (Figure 2).

In summary, these results supported the feasibility of our combined *in silico* and proteomic-based approach to identify new biomarkers for *Brucella* diagnostics that enable the differentiation between a *B. melitensis* reference strain and a closely related vaccine strain.

### Differentiation of *Brucella melitensis* Field Isolates From the *Brucella melitensis* Rev.1 Vaccine Strain

Subsequently, we assessed the robustness of the discrimination between *B. melitensis* wild-type isolates and the *B. melitensis* Rev.1 vaccine strain based on the ribosomal proteins L24 and S12. For this purpose, we analyzed *B. melitensis* isolates collected from humans, goats, sheep and cattle in Israel, where ovine and caprine brucellosis is known to be endemic and a routine vaccination program for livestock with *B. melitensis* Rev.1 has been implemented during the last decades (Banai, 2002). Moreover, the *B. melitensis* strains endemic in Israel mostly belong to the MLVA group “*melitensis* East-Mediterranean,” whereas *B. melitensis* Rev.1 is part of the MLVA group “*melitensis* Americas” (Vergnaud et al., 2018).

The MALDI-TOF MS spectra of 18 *B. melitensis* Rev.1 vaccine strains and 183 Israeli *B. melitensis* field isolates were compared, and we further evaluated the impact of spectra acquisition by performing MALDI-TOF MS measurements at the two institutes BfR and KVI (Supplementary Figure 1). As seen before in the comparison between *B. melitensis* Rev.1 and *B. melitensis* 16M (Figure 2), we detected a distinct peak pair in the sample spectra for the ribosomal protein L24 with averaged accurate masses at *m/z* 11,178 in the vaccine strain group “*melitensis* Rev.1” and *m/z* 11,208 in the field strain group “*melitensis*” (Figure 3). In the mass range for double charged ions of the ribosomal protein L24, we detected accurate masses at *m/z* 5,590 (group “*melitensis* Rev.1”) and *m/z* 5,605 (group “*melitensis”*) (Supplementary Figure 2) corresponding to the ions [M_*Thr28*_+2H]^2+^ and [M_Met28_+2H]^2+^, respectively. In a subset of “*melitensis*” field strains samples, this peak pair is interspersed with a second signal at *m/z* 5,595, which is not present in the single charged state (Supplementary Figure 2B, “*melitensis*” groups).

Furthermore, the peak pair for the ribosomal protein S12 was found in the sample spectra with averaged accurate masses at *m/z* 13,784 in the field strain group “*melitensis*” and *m/z* 13,798 in the vaccine strain group “*melitensis* Rev.1” (Supplementary Figure 3). The exact mass difference of +16.0 Da between the ion [M_Pro91_+H]^+^*_*melitensis*_* with *m/z* 13,871.2 and the ion [M_Leu91_+H]^+^*_*melitensis Rev.1*_* with *m/z* 13,887.2 corresponded well to the observed peak shift. However, for peaks that were not within the mass window of 3,000 to 11,500 Da (defined by reference peaks with at least 90% frequency in all measurements, see Supplementary Table 4), the alignment by a locally weighted scatterplot smoothing function (LOWESS) was less accurate, leading to a higher mass scattering. Ribosomal protein S12 ion variants show respective peaks in the “BfR” subgroups with the strongest intensities for ion [M-Met+βMeS+H]^+^, the beta-methylthiolated ribosomal protein S12 ion lacking the N-terminal methionine (Supplementary Figure 3). In the mass range for double charged ions of probable ribosomal protein S12, we detected accurate masses at *m/z* 6,894 (group “*melitensis*”) and *m/z* 6,902 (group “*melitensis* Rev.1”) (Supplementary Figure 4) corresponding to the ions [M_Pro91_-Met+βMeS+2H]^2+^ and [M_Leu91_-Met+βMeS+2H]^2+^, respectively.

Based on our peak observations, we determined mass windows with a decision boundary to distinguish between ribosomal protein L24 and S12 variants, and tested whether these thresholds facilitate the differentiation between the *B. melitensis* field isolates and the Rev.1 vaccine strain (Table 2). The alignment procedure against a reference spectrum decreased the standard deviation considerably (Table 2), e.g., for “BfR” peaks within the ribosomal protein L24 (z = 1) decision mass window (11,197.5:11,235) from mean_non–aligned_ = 11,206.9 with SD_non–aligned_ = 2.9 for technical replicate spectra to mean_aligned_ = 11,208.1 with SD_aligned_ = 0.4 for sample spectra (Figures 3D–F). Peaks from the KVI measurement scattered more widely (technical replicate spectra mean_non–aligned_ = 11,213.3 with SD_non–aligned_ = 4.7 versus sample spectra mean_aligned_ = 11,211.4 with SD_aligned_ = 4.5), due to a recalibration of the KVI instrument between measurements.

Recently, a study has been published that also aimed to detect biomarkers enabling the discrimination of *B. melitensis* field isolates and the Rev.1 vaccine strain by MALDI-TOF MS (Christoforidou et al., 2020). For this purpose, 73 clinical and veterinary *B. melitensis* isolates from Greece were analyzed. Initially, a cluster analysis on a subset of 17 field strains that represented the three *B. melitensis* biovars against three commercial *B. melitensis* Rev.1 strains had been performed. Two discriminating peaks were described in this study: Peak *m/z* 3,528 could be detected in all tested Greek *B. melitensis* field isolates but not in *B. melitensis* Rev.1, whereas peak *m/z* 7,328 was unique for the vaccine strain (Christoforidou et al., 2020). Strikingly, these biomarker peaks were not identified in our study, thus we compared the data published by Christoforidou et al. (2020) with our data. The first described biomarker with *m/z* 3,528, supposed to be only present in the spectra of *B. melitensis* Greek field isolates, could be found in the spectra of all Israeli field isolates, but also in the *B. melitensis* 16M reference strain and the Rev.1 strain (Figure 4A). The second biomarker with *m/z* 7,328 neither occurred in the Greek *B. melitensis* field isolates (Christoforidou et al., 2020) nor in the *B. melitensis* field isolates from Israel (Figure 4A), but in the *B. melitensis* Rev.1 strain of both studies. However, this peak does not reflect a true biomarker for *B. melitensis* Rev.1 against the closely related *B. melitensis* 16M from the same MLVA group “*melitensis* Americas,” which also harbors this causative protein (Figure 4A).

In contrast, our here reported ribosomal protein biomarkers L24 and S12 distinguish the *B. melitensis* Rev.1 vaccine strain not only from the Israeli *B. melitensis* field isolates but also from *B. melitensis* 16M (Figure 4B). Christoforidou et al. (2020) probably did not detect these ribosomal biomarkers since their forward analysis tolerance parameter, which was not stated, may have excluded the detection of close peaks with the Mass-Up default settings or due to low mass intensities in *m/z* ranges above 10 kDa. In order to prove the universal character of our biomarkers and to exclude clonal and regional effects, *B. melitensis* strains from 17 different MLVA groups (Supplementary Table 6) were subjected to MALDI-TOF MS analysis and the spectra were searched for the presence of the protein biomarkers L24 and S12. While the biomarker peaks described by Christoforidou et al. (2020) for the differentiation of *B. melitensis* Rev.1 could also be found in the *B. melitensis* field strains of this diversity set (Figure 4C), the analysis confirmed the discriminatory power of the L24 and S12 biomarkers, which distinguish the *B. melitensis* Rev.1 vaccine strain from naturally occurring *B. melitensis* strains in general (Figure 4D).

### Discriminatory Power of the L24 and S12 Signals for the Strain-Level Identification of *Brucella melitensis* Rev.1 by MALDI-TOF MS

We further evaluated the uniqueness of the ribosomal proteins L24 and S12 as marker proteins for the unambiguous identification of *B. melitensis* Rev.1 by comparing their amino acid sequences with the L24 and S12 protein entries in the NCBI protein database originating from 802 sequenced *Brucella* isolates.

Unexpectedly, a blastP-based comparison did not identify a protein sequence identical to the L24-M28T variant of *B. melitensis* Rev.1 in any other isolate of the NCBI genome dataset (Figure 5 and Supplementary Table 7). Instead, the amino acid sequences of the ribosomal L24 proteins from most isolates of the analyzed *Brucella* species were identical to the L24 protein sequence of *B. melitensis* 16M. However, few isolates of the *B. abortus* clade C, of the *B. melitensis* East-Mediterranean clade and of *B. suis* bv. 2 harbor altered L24 protein sequences with the amino acid exchanges K90R, N67S and G55D, respectively (Figure 6). The L24 protein variant of the *B. melitensis* East-Mediterranean isolate with the N67S amino acid exchange exhibits a −27 Da mass difference to the wild-type L24 protein. Hence, its spectrum peak cannot be distinguished by MALDI-TOF MS from the −30 Da spectrum peak of L24 variant from *B. melitensis* Rev.1 (Supplementary Table 7). Interestingly, all *B. ovis* isolates encode for a L24 protein with the unique modification V81I resulting in a +14 Da mass difference compared to the common L24 proteins (Figure 5 and Supplementary Table 7).

For ribosomal protein S12, *B. melitensis* Rev.1 exclusively carried the P91L mutation with its +16 Da mass difference compared to the most frequent S12 variant in the NCBI dataset (Figure 5 and Supplementary Table 8). However, the ribosomal S12 proteins of all analyzed *B. ceti* clade B isolates harbor the amino acid exchange V55I that leads to a mass difference of +14 Da. Consequently, this S12 protein variant is indistinguishable from the corresponding S12 protein peak of *B. melitensis* Rev.1 in MALDI-TOF MS analysis. Moreover, few strains of the *B. abortus* clade C encode for a ribosomal S12 protein with a K43R exchange and a limited number of isolates of the *B. melitensis* West-Mediterranean clade express S12 protein variants with the amino acid changes K43R or K88R (Figure 6 and Supplementary Table 8). Mutations at corresponding codon positions have been identified in streptomycin-resistant strains of other bacterial species (Supplementary Figure 5) like *Escherichia coli*, *Salmonella* Typhimurium, *Mycobacterium tuberculosis*, *Helicobacter pylori*, *Klebsiella pneumoniae*, *Erwinia carotovora* or *Thermus thermophiles* (Finken et al., 1993; Bjorkman et al., 1999; Gregory et al., 2001; Torii et al., 2003; Chumpolkulwong et al., 2004; Barnard et al., 2010; Tsai et al., 2014).

Taken together, our bioinformatics analysis suggests that the ribosomal protein variants of L24 and S12 identified here as biomarkers for *B. melitensis* Rev.1 have extremely high discriminatory properties allowing the direct differentiation of this vaccine strain from other *Brucella* isolates even without performing a preceding classification as *B. melitensis*.

## Discussion

The worldwide emerging zoonotic disease brucellosis affects wildlife and livestock, especially cattle, goats and sheep (Seleem et al., 2010; Godfroid, 2017). In countries with endemic brucellosis, vaccination programs have been undertaken to combat *Brucella* infections in farm animals (Banai, 2002; Olsen and Stoffregen, 2005). However, these measures require efficient diagnostic tools to distinguish vaccine strains from naturally occurring wild-type *Brucella* strains in the livestock herds.

Here, we propose MALDI-TOF MS profiling as a cost-effective alternative to the currently applied methods of classical microbiological testing (Elberg and Meyer, 1958) and molecular PCR-techniques (Bardenstein et al., 2002; Lopez-Goni et al., 2008; Alvarez et al., 2017; Christoforidou et al., 2018) for the differentiation of the *B. melitensis* Rev.1 vaccine strain from *B. melitensis* field isolates. This approach will be of benefit for reference laboratories and large healthcare facilities that have already implemented MALDI-TOF MS diagnostics for the identification of bacterial pathogens. Moreover, due to the robustness of mass spectrometry, this rapid identification method is increasingly used in tropical countries, where it complements smaller point-of-care laboratories (Fall et al., 2015; Chabriere et al., 2018).

Whole genome comparison has previously identified various mutations as genetic markers that distinguish *B. melitensis* Rev.1 from reference strain *B. melitensis* 16M (Issa and Ashhab, 2016). However, besides a non-synonymous mutation affecting the ribosomal protein S12, most of the 32 listed genome-specific markers were not identified by our *in silico* analysis or did not translate into changes of MALDI-TOF MS peaks. This might be the consequence of (i) our filter criteria that restricted potential marker proteins on masses between 2,000 and 20,000 Da, (ii) mutations that do not translate into amino acid exchanges captured by MALDI-TOF MS, (iii) insufficient *in vitro* expression of the marker genes, or (iv) MALDI-TOF MS signal suppression effects leading to undetectable amounts of protein.

Our comparative *in silico* analysis of the translated open reading frames from the genomes of *B. melitensis* 16M and Rev.1 predicted 17 potential MALDI-TOF MS biomarkers for the differentiation of both strains. Subsequent whole-cell MALDI-TOF MS spectra comparison of *B. melitensis* 16M and Rev.1 confirmed that two marker peak pairs, resembling the ribosomal proteins L24 and S12 in their single and double charged states, have the discriminatory power to distinguish between these closely related *B. melitensis* strains. Our observation is in agreement with previous studies, demonstrating that the sequence variabilities and abundance of ribosomal proteins in microbial cells allow their usage as robust biomarkers for the identification of pathogenic and non-pathogenic bacteria at species and strain level by whole-cell MALDI-TOF MS (Sandrin et al., 2013).

The ribosomal protein L24 is encoded by the *rplX* gene, for which spontaneous missense mutations have been described before (Nishi et al., 1987; Sharp et al., 1992). Accordingly, our comparative bioinformatics analysis of the *Brucella* pan proteome not only identified the altered L24 protein in *B. melitensis* Rev.1 but also five additional variants. These mutated L24 proteins exhibit mass differences from about −30 to +69 Da compared to the wild-type L24 protein of *Brucella* and may represent additional biomarkers, especially for the identification of *Brucella ovis* and for the improved differentiation of *Brucella* and closely related former *Ochrobactrum* species. Likewise, MALDI-TOF MS studies of various other bacteria have identified peak differences of L24 protein variants. Consequently, the ribosomal protein L24 has served as species-specific biomarker for *Flavobacterium psychrophilum*, *Bacillus* spp., *Streptococcus thermophilus*, *Listeria monocytogenes* and *Francisella tularensis* as well (Teramoto et al., 2007; Hotta et al., 2011; Durighello et al., 2014; Ojima-Kato et al., 2016; Fernandez-Alvarez et al., 2018).

The streptomycin resistance is a distinct characteristic of vaccine strain *B. melitensis* Rev.1 that was introduced during vaccine derivation by Elberg and Faunce (1957). It is mediated through a spontaneous *rpsL* mutation that leads to an altered ribosomal protein S12 with the amino acid exchange Pro to Leu at codon position 91 (P91L) (Cloeckaert et al., 2002). We showed here that this altered protein sequence resulted in a distinct shift of the MALDI-TOF MS peak *m/z* 13,784, which can be used as a biomarker for the identification of *B. melitensis* Rev.1.

Two post-translational modifications of ribosomal protein S12 have been observed in other bacterial species (Kowalak and Walsh, 1996; Suh et al., 2005) or were inferred from sequence similarity: NME (average mass change: −131.2 Da) and beta-methylthiolation of Asp89 (average mass change: +46.1 Da, see UniProt annotation of RS12_BRUME). We also found this mutation in the *rpsL* genes of streptomycin resistant *T. thermophilus*, *K. pneumoniae* and *E. coli* isolates, further illustrating that certain antibiotic resistances in bacteria correlate with ribosomal protein changes that can be detected by MALDI-TOF MS (Wilcox et al., 2001; Carr et al., 2005).

The usage of the ribosomal proteins S12 and L24 signals as MALDI-TOF MS biomarkers enabled the unequivocal discrimination between *B. melitensis* Rev.1 and the analyzed Israeli *B. melitensis* field isolates, as well as the closely related type strain *B. melitensis* 16M. Comparable results were obtained from MALDI-TOF MS measurements performed at two different institutes, emphasizing the high quality, accuracy and reproducibility of the established method.

Moreover, our bioinformatics analysis of hitherto published *Brucella* proteomes did not identify any other *Brucella* isolate that encodes for S12 and L24 proteins with identical amino acid sequences, further illustrating their unique character. Our here identified biomarkers for the identification of the *B. melitensis* Rev.1 vaccine strain had not been identified by a recent MALDI-TOF MS based study pursuing the same aim (Christoforidou et al., 2020). The two alternative biomarkers described in the work by Christoforidou et al. (2020) for the discrimination between *B. melitensis* field isolates and the Rev.1 vaccine strain may only be of benefit for local applications in Greece. According to our analysis, the first proposed biomarker (*m/z* 3,528) was not only present in *B. melitensis* field isolates and *B. melitensis* 16M, but also in *B. melitensis* Rev.1, whereas the second putative biomarker (*m/z* 7,328) was not exclusive for *B. melitensis* Rev.1 as indicated, but was also found in *B. melitensis* 16M. Two analytical restraints in their study could have contributed to these different findings. First, the mass trimming to a window of interest has omitted the detection of the single charged variants of probable ribosomal proteins L24 and S12. Second, mathematical binning of continuous sample peak *m/z* values for subsequent cluster analysis utilized a tolerance threshold that limited its discriminatory power. The double charged ribosomal protein L24 variants in our study were detectable at a mass resolution of 400 whereas double charged ribosomal protein S12 variants required a higher analytical mass resolution of 860. Christoforidou and colleagues used the MALDIquant default tolerance of 0.002 for peak binning, i.e., a mass resolution limit of 250, and therefore, were not able to detect any of the double charged variants of ribosomal protein L24 or S12. Furthermore, if spectra within a group display diversity, as seen in the ribosomal protein L24 subset of “*melitensis*” field isolates at *m/z* 5,595 (Supplementary Figure 2), biomarker significance in statistical tests will diminish. Applying these analytical considerations, our MALDI-TOF MS approach identified two genus-wide unique biomarkers that unambiguously discriminated *B. melitensis* Rev.1 from *B. melitensis* field isolates and the closely related type strain *B. melitensis* 16M without any misidentification.

Several manufacturers around the world produce the *B. melitensis* Rev.1 vaccine strain from different seed stocks. Hence, a comprehensive characterization of *B. melitensis* Rev.1 is required for quality controls in vaccine production facilities, since Rev.1 strains of different production sites may differ significantly from the original Elberg strain (Bosseray, 1991). To avoid alterations in the attenuated virulence and to assure strain stability during the vaccine production process, the gene expression profile of the *B. melitensis* Rev.1 strain should be checked on a regular basis. In this context, our MALDI-TOF MS based analysis may serve as an additional standardization tool in the commercial production of the *B. melitensis* Rev.1 vaccine.

## Conclusion

The here described ribosomal marker proteins for distinguishing the vaccine strain *B. melitensis* Rev.1 from *B. melitensis* field strains by MALDI-TOF MS will improve the differential diagnosis necessary for brucellosis control efforts within vaccination programs and subsequently the successful eradication of the zoonoses from small ruminants. Natural streptomycin resistance in *Brucella* is rare, but *Brucella* species exhibit variable streptomycin susceptibilities and the development of spontaneous resistance has been seen *in vitro* (Hall and Manion, 1970; Hall, 1990). This observation is supported by our identification of *Brucella* isolates that encode for ribosomal protein S12 variants with amino acid sequences of streptomycin resistant alleles. Our approach does not detect streptomycin resistance mediated by mutations of the 16S rRNA (Springer et al., 2001). However, the improved MALDI-TOF MS based screening for the *B. melitensis* Rev.1 vaccine strain and other streptomycin resistant *B. melitensis* field isolates with ribosomal protein S12 variants may reduce the risk of an inadequate first line therapy in human brucellosis, since streptomycin is commonly used in the antibiotic regimen applied for patients infected with *Brucella* (Skalsky et al., 2008; Meng et al., 2018).

## Data Availability Statement

Most of the datasets presented in this study can be found in online repositories. The names of the repository/repositories and accession number(s) can be found in the article/Supplementary Material, further inquiries can be directed to the corresponding authors.

## Author Contributions

HB, DK, DH, and SAD: conceptualization and writing—review and editing. HB, DK, and DH: data curation and writing—original draft. HB: formal analysis, methodology, software, and visualization. SAD: funding acquisition, project administration, and supervision. HB, DK, and SM: investigation. SEB, MF, and SB: resources. HB, DK, DH, SAD, MF, SEB, and SM: validation. All authors contributed to the article and approved the submitted version.

## Conflict of Interest

The authors declare that the research was conducted in the absence of any commercial or financial relationships that could be construed as a potential conflict of interest.

## Publisher’s Note

All claims expressed in this article are solely those of the authors and do not necessarily represent those of their affiliated organizations, or those of the publisher, the editors and the reviewers. Any product that may be evaluated in this article, or claim that may be made by its manufacturer, is not guaranteed or endorsed by the publisher.

## Footnotes

1
www.ncbi.nlm.nih.gov/assembly

2
www.patricbrc.org

3
https://www.ncbi.nlm.nih.gov/assembly/?term=txid234[Organism:exp]

4
https://github.com/tseemann/mlst

5
https://microbesgenotyping.i2bc.paris-saclay.fr
