# Supplementary material for: Protein Biomarker Identification for the Discrimination of Brucella melitensis Field Isolates From the Brucella melitensis Rev.1 Vaccine Strain by MALDI-TOF MS
Source: Front Microbiol. 2021 Oct 22;12:712601. doi: 10.3389/fmicb.2021.712601 (PMC8569450; doi:10.3389/fmicb.2021.712601)
Supplement: Supplementary file 1 [file Data_Sheet_1.docx]

Supplementary Material

# Supplementary Figures





**Supplementary Figure 1 |** Gel depiction of sample spectra. Samples were sorted (A) alphabetically or (B) by Ward’s hierarchical clustering algorithm based on an absence/presence feature matrix of binned peaks (binning tolerance: 0.0008). Note: MOAG is another abbreviation for the institute KVI.


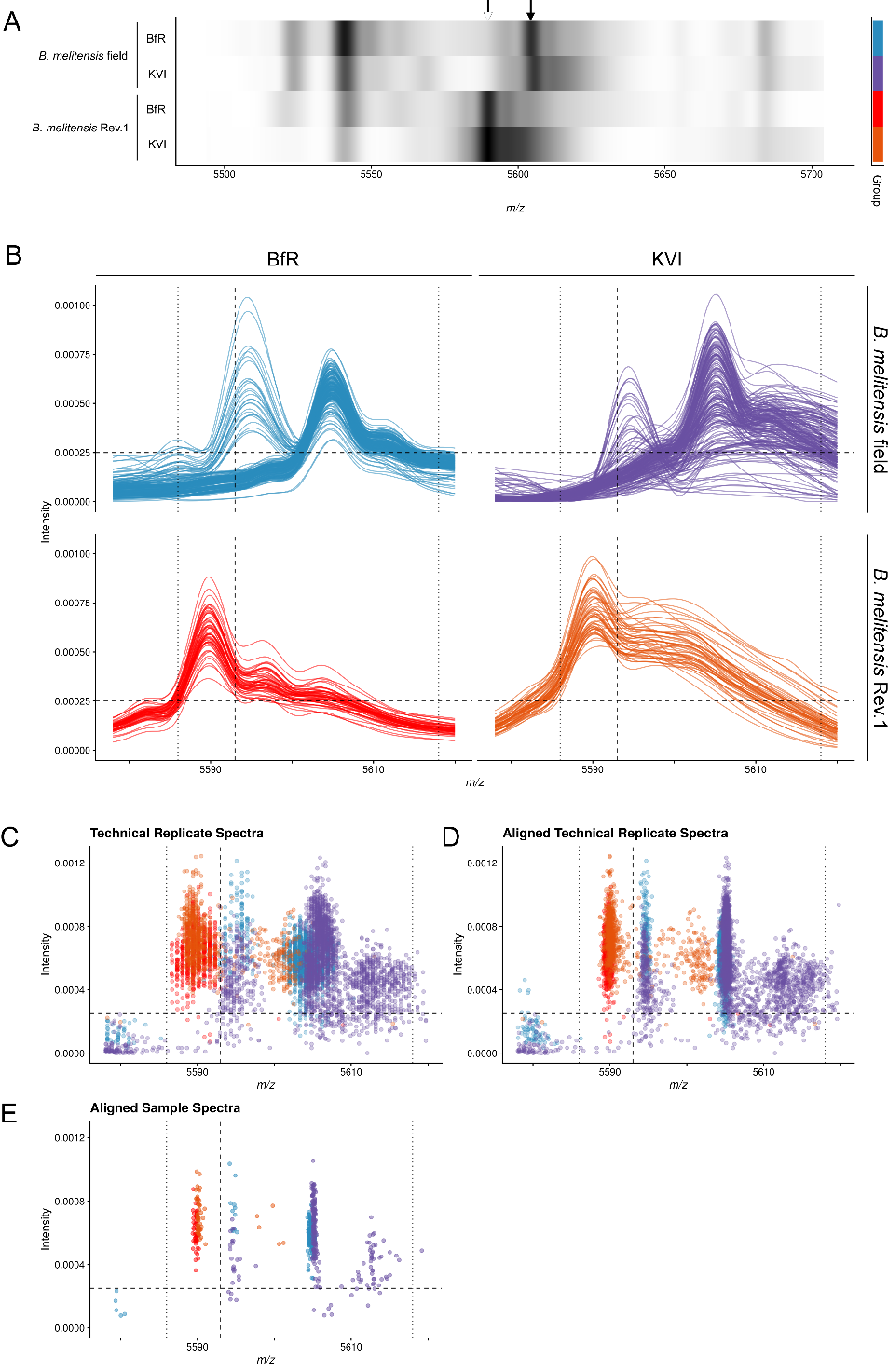


**Supplementary Figure 2 |** Peak discrimination for the double charged ribosomal protein L24. (A) Gel view of group spectra derived from the field isolate group “*melitensis*” and the vaccine strain group “*melitensis* Rev.1” presented by institute “BfR” and “KVI”, magnified to the *m/z* range enclosing the exact masses of PLF_234_00001168, z=2. Arrows point to accurate masses at 5590 Da (white, [M_Thr28_+2H]^2+^ ion) and 5605 Da (black, [M_Met28_+2H]^2+^ ion). (B) Line graphs of aligned sample spectra shown by group and institute. (C-E) Scatterplot of peaks in the technical replicate spectra before (C) and after alignment (D) against the reference spectrum. (E) Peaks derived from aligned sample spectra. Intensity cut-off (horizontal dashed line) in panels B-E: 0.00025 AU; for mass cut-offs (decision window: vertical dotted lines, decision boundary: vertical dashed line) see Table 2.


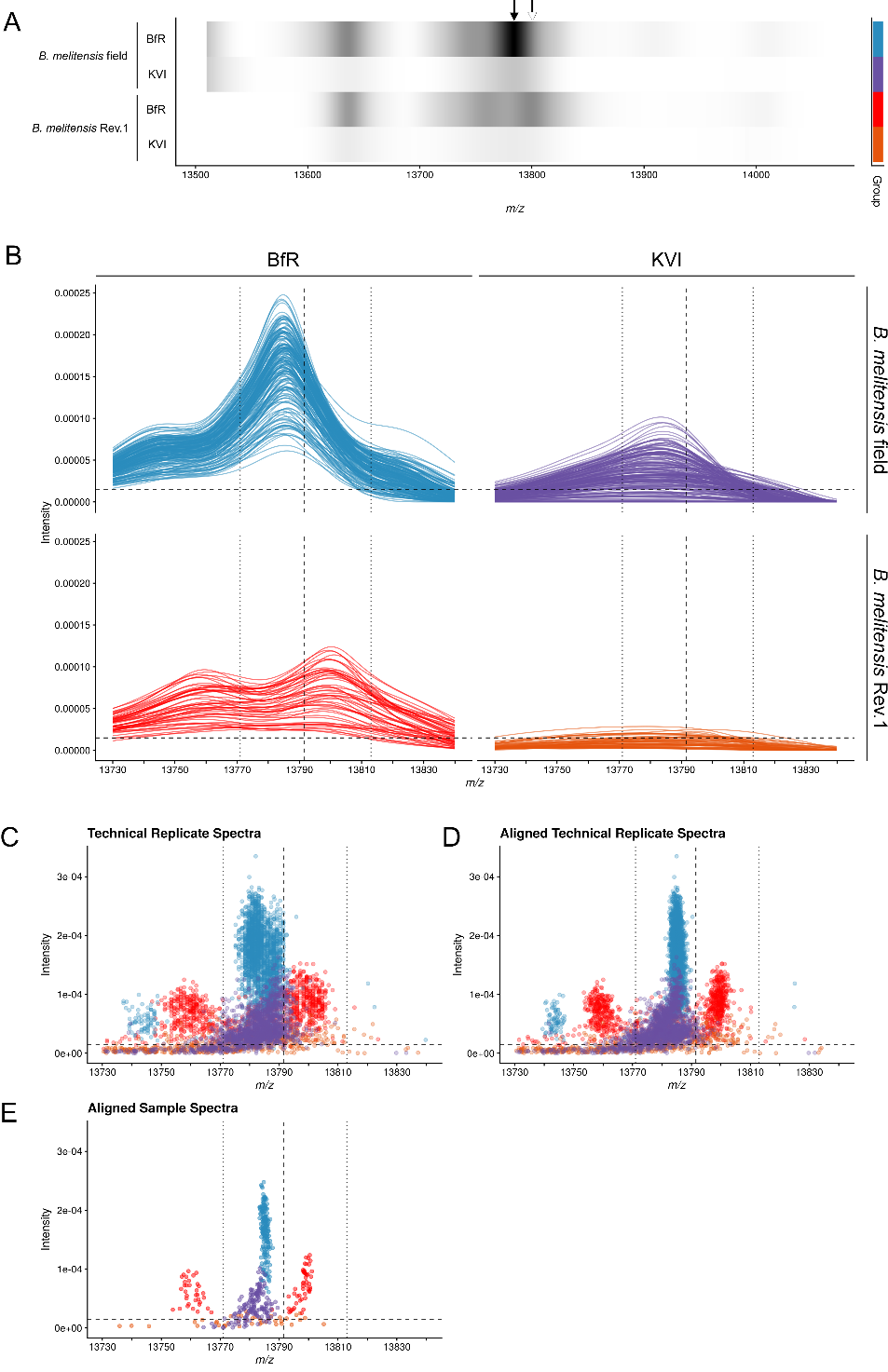


**Supplementary Figure 3 |** Peak discrimination for the single charged ribosomal protein S12. (A-E) For content description, refer to Figure S2. *m/z* range for PLF_234_00002366, z=1. Arrows point to accurate masses at 13784 Da (black, [M_Pro91_‑Met+βMeS+H]^+^ ion) and 13798 Da (white, [M_Leu91_‑Met+βMeS+H]^+^ ion). Intensity cut-off (horizontal dashed line) in panels B-E: 0.000015 AU; for mass cut-offs (decision window: vertical dotted lines, decision boundary: vertical dashed line) see Table 2.


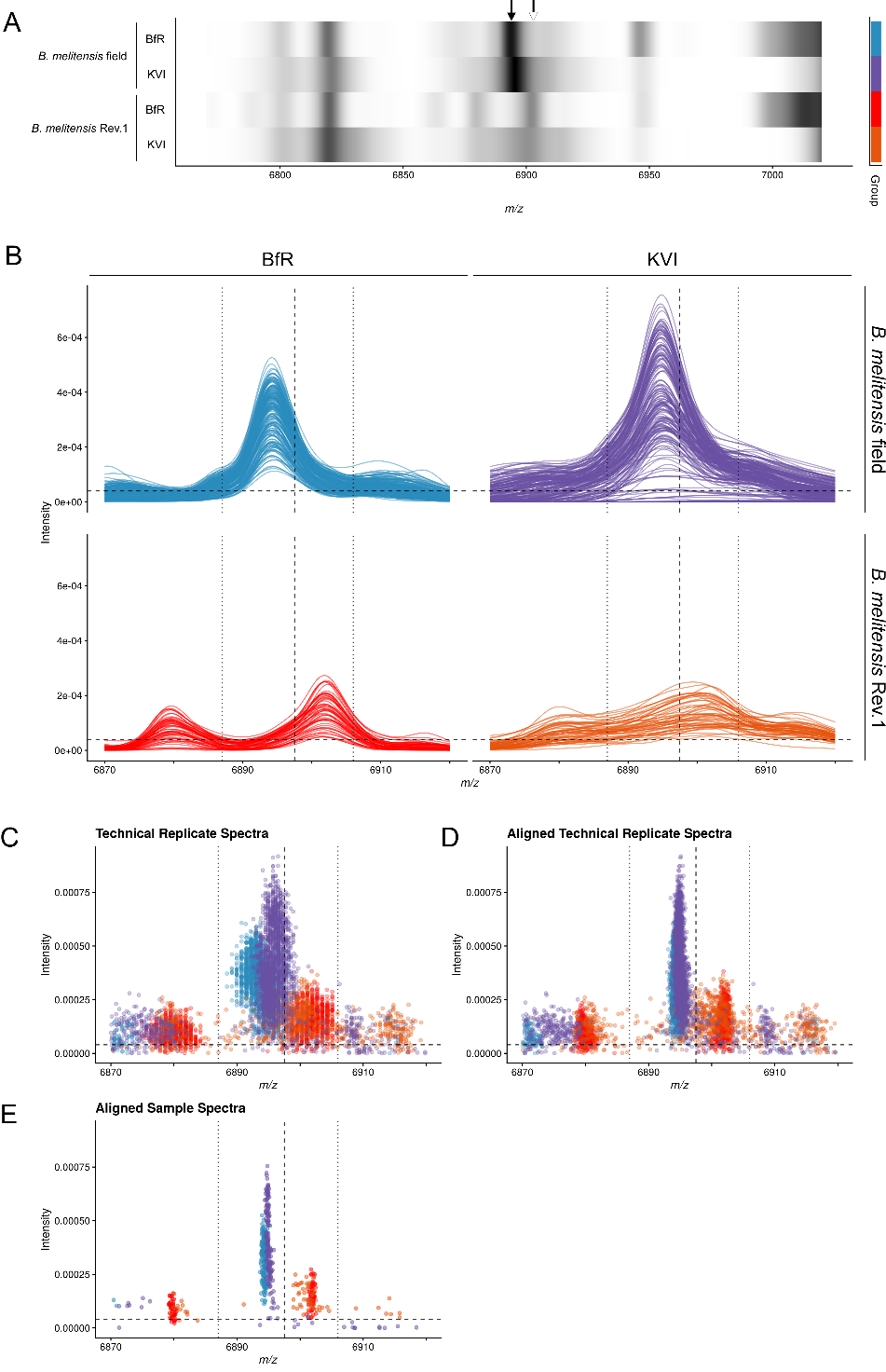


**Supplementary Figure 4 |** Peak discrimination for the double charged ribosomal protein S12. (A-E) For content description, refer to Figure S2. *m/z* range for PLF_234_00002366, z=2. Arrows point to accurate masses at 6894 Da (black, [M_Pro91_‑Met+βMeS+2H]^2+^ ion) and 6902 Da (white, [M_Leu91_‑Met+βMeS+2H]^2+^ ion). Intensity cut-off (horizontal dashed line) in panels B-E: 0.00004 AU; for mass cut-offs (decision window: vertical dotted lines, decision boundary: vertical dashed line) see Table 2.


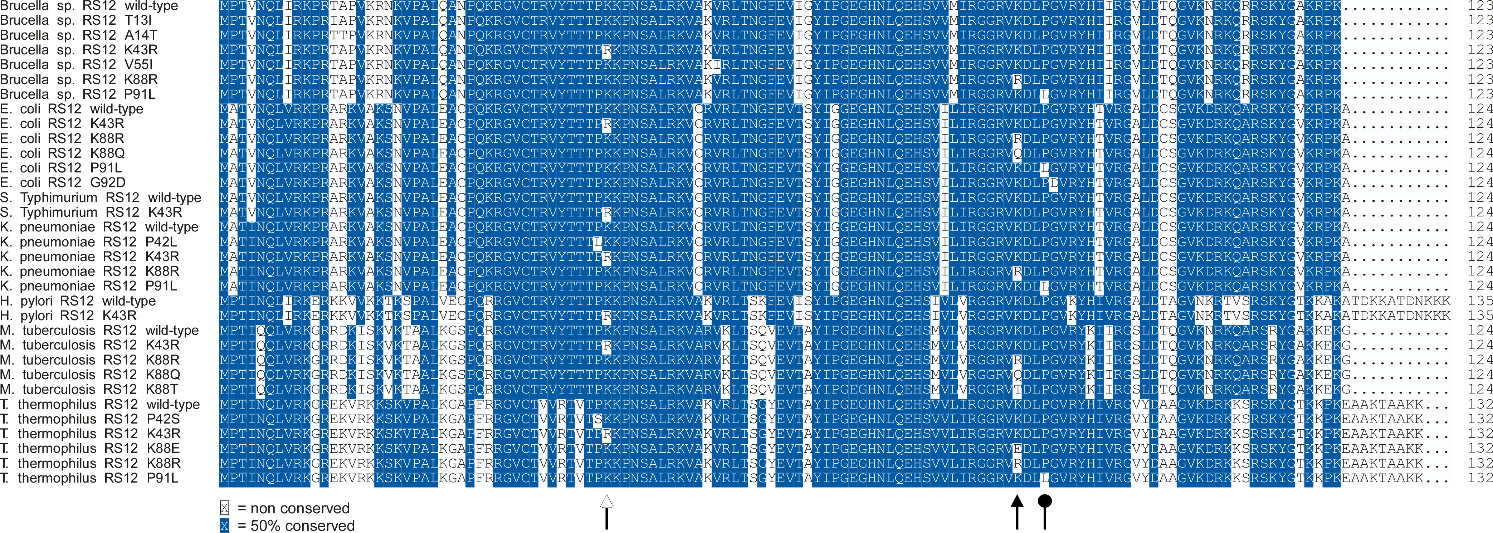


**Supplementary Figure 5 |** Multiple amino acid sequence alignment of selected ribosomal protein S12 variants. The variants K43R (white arrow), K88R (black arrow) and P90L (circle arrow) are known determinants for streptomycin resistance in the listed bacteria. Note: Amino acid numbering starts from the initial methionine and does not consider NEM; in contrast, the *E. coli* numbering scheme starts with the alanine at position 2.

# Supplementary Tables

Note: Supplementary Tables are provided as separate Microsoft Excel files.

Supplementary Table 1 | The PATRIC-RASTtk annotation feature tables for the strains *B. melitensis* 16M and *B. melitensis* Rev.1.

Supplementary Table 2 | Mass divergence between homologous proteins of *B. melitensis* 16M and *B. melitensis* Rev.1.

Supplementary Table 3 | Sample metadata.

Supplementary Table 4 | Reference peaks.

Supplementary Table 5 | *In silico* molecular typing results.

Supplementary Table 6 | *Brucella melitensis* strains in the diversity set and their MLVA8 genotype.

Supplementary Table 7 | L24 protein variant frequency.

Supplementary Table 8 | S12 protein variant frequency.
